# Time-Dependent Demineralization of Tilapia (*Oreochromis niloticus)* Bones Using Hydrochloric Acid for Extracellular Matrix Extraction

**DOI:** 10.3390/biomimetics8020217

**Published:** 2023-05-23

**Authors:** Michael John Nisperos, Hernando Bacosa, Gladine Lumancas, Fernan Arellano, Jemwel Aron, Lean Baclayon, Zesreal Cain Bantilan, Marionilo Labares, Ronald Bual

**Affiliations:** 1 Environmental Science Graduate Program, Department of Biological Sciences, College of Science and Mathematics, Mindanao State University-Iligan Institute of Technology, Iligan City 9200, Philippines; michaeljohn.nisperos@g.msuiit.edu.ph (M.J.N.); hernando.bacosa@g.msuiit.edu.ph (H.B.); gladine.lumancas@g.msuiit.edu.ph (G.L.); fernan.arellano@g.msuiit.edu.ph (F.A.); jemwel.aron@g.msuiit.edu.ph (J.A.); lean.baclayon@g.msuiit.edu.ph (L.B.); 2 Center for Sustainable Polymers, Mindanao State University-Iligan Institute of Technology, Iligan City 9200, Philippines; zesrealcain.bantilan@g.msuiit.edu.ph (Z.C.B.);; 3 Department of Chemical Engineering and Technology, College of Engineering, Mindanao State University-Iligan Institute of Technology, Iligan City 9200, Philippines

**Keywords:** tilapia, fish bones, demineralization, extracellular matrix, kinetics

## Abstract

Tilapia *(Oreochromis niloticus*) is a widely cultivated fish in tropical and subtropical regions such as the Philippines, generating substantial waste during processing, including bones that are a valuable source of extracellular matrix (ECM). However, the extraction of ECM from fish bones requires an essential step of demineralization. This study aimed to assess the efficiency of tilapia bone demineralization using 0.5 N HCl at different time durations. By evaluating the residual calcium concentration, reaction kinetics, protein content, and extracellular matrix (ECM) integrity through histological analysis, composition assessment, and thermal analysis, the effectiveness of the process was determined. Results revealed that after 1 h of demineralization, the calcium and protein contents were 1.10 ± 0.12% and 88.7 ± 0.58 μg/mL, respectively. The study found that after 6 h, the calcium content was almost completely removed, but the protein content was only 51.7 ± 1.52 μg/mL compared to 109.0 ± 1.0 μg/mL in native bone tissue. Additionally, the demineralization reaction followed second-order kinetics with an R^2^ value of 0.9964. Histological analysis using H&E staining revealed a gradual disappearance of the basophilic components and the emergence of lacunae, which can be attributed to decellularization and mineral content removal, respectively. As a result, organic components such as collagen remained in the bone samples. ATR-FTIR analysis showed that all demineralized bone samples retained collagen type I markers, including amide I, II, and III, amides A and B, and symmetric and antisymmetric CH_2_ bands. These findings provide a route for developing an effective demineralization protocol to extract high-quality ECM from fish bones, which could have important nutraceutical and biomedical applications.

## 1. Introduction

In tropical and subtropical regions, tilapia is a popular fish species for cultivation due to its resilience and adaptability to various environmental conditions. In the Philippines, the aquaculture industry has experienced significant growth over the past few decades, with tilapia production alone reaching approximately 304,326.59 MT in 2020 [1]. However, this increased production also leads to the generation of a significant amount of waste, including viscera, skin, scales, and bones which, if not appropriately disposed of, can cause eutrophication, oxygen depletion, and the release of toxic compounds into aquatic ecosystems [2]. These wastes could also contribute to greenhouse gas emissions and other environmental problems if disposed of in landfills. Therefore, it is crucial to make the most of these resources to unlock their full potential in creating high-value products.

Despite increasing efforts to utilize fish industry waste for new products, most waste is still being used for low-value applications. These typical applications include, but are not limited to, animal and aquaculture feeds [3], energy production through biogas and methane [4], and fish glue [5]. However, fish waste can also be converted into a product with a high added value [6,7], particularly by extracting marine-based extracellular matrix (ECM) from tilapia bones. More importantly, it also aligns with the United Nations Sustainable Development Goal 12: Ensure Sustainable Consumption and Production Patterns, which targets substantially reducing waste generation, and it is also a noble process in transitioning to a circular economy.

Collagen, one of the primary constituents of the extracellular matrix (ECM), plays a crucial role in providing mechanical support to tissues and organs and regulating the cellular environment [8,9,10,11]. In bone tissue, collagen makes up about 90% of the ECM, with the remaining 10% consisting mainly of non-collagenous proteins and proteoglycans [12,13,14]. Collagen is typically harvested from the vertebrae of swine and bovine animals, which poses the risk of contracting an animal disease that is likely to be transmitted to humans [15,16]. In addition, due to religious convictions, Jews and other religious groups do not consume any foods derived from swine and bovine animals [15,17]. Marine-based collagen type I has been shown to have several advantages over its land-based counterparts, including higher bioavailability, water solubility, and improved stability, and its derivatives have proven beneficial to bone-related diseases such as osteoporosis and osteoarthritis [18], which makes it highly sought after for nutraceutical applications.

Extracting the ECM structure, mainly composed of collagen type I, from bones can be exceedingly difficult. This is due to collagen being deposited within the hydroxyapatite (HA) crystals [(Ca)_10_(PO_4_)_6_(OH)_2_] that makes up the bone’s structure forming a fibrous scaffold [19]. This makes bone demineralization a necessary step in the process of extracting collagen. Pang et al. studied four commonly used demineralizing agents: ethylenediaminetetraacetic acid (EDTA), formic acid (CH_2_O_2_), hydrochloric acid (HCl), and HCl/EDTA mixture. Their results showed that demineralization for 7 h using HCl yielded optimal mineral removal, such as calcium [20].

HCl as a demineralizing agent reacts efficiently with hydroxyapatite, the major inorganic constituent of bone, to form monocalcium phosphate and calcium chloride, both of which are water-soluble and easily removed [21]. Bone samples are typically demineralized in 0.5–0.6 M HCl to achieve efficient mineral removal while preserving graft osteoinductive potential [22]. However, the demineralization process can also impact the structural and mechanical properties of the bone matrix. Therefore, the demineralization protocol must be studied for the intended use of the demineralized bone matrix to ensure that its properties are appropriate for the desired application.

Hence, this study aimed to examine the optimal demineralization conditions using 0.5 M HCl and evaluate the physical, chemical, and thermal properties of the resulting demineralized bone. The characteristics of the demineralized bone provide valuable insights into its potential functional properties for use in the nutraceutical and biomedical industries. Furthermore, our research offers a sustainable solution to tilapia processing waste and promotes a circular economy, benefiting the tilapia aquaculture industry. The study creates new market opportunities while reducing waste, contributing to a more sustainable and eco-friendly society.

## 2. Materials and Methods

### 2.1. Preparation and Demineralization of Tilapia Bone

The tilapia fish were procured from a local fish landing and were processed immediately to extract the vertebrae. The samples were subjected to rigorous cleaning to ensure the preservation of the specimens. First, the samples were segmented per vertebra (average length of 0.5 cm) and washed meticulously with distilled water. Subsequently, the bone segments were stirred in phosphate-buffered saline (1X PBS, pH 7.4) solution for a period of 2 h. Finally, the bones were gently patted dry to remove excess moisture.

The demineralization was carried out at room temperature using 0.5 N hydrochloric acid (HCl) as the demineralizing agent. The demineralization process involved a solvent to mass ratio of 25:1 (mL of HCl: g of tilapia bones) and stirring speed of 300 rpm using a magnetic stirrer (HCS M60-Pro, Singapore). The demineralization times used were 5 min, 10 min, 30 min, 1 h, 6 h, 12 h, and 24 h. With 7 different processing times and 3 replicates, a total of 21 samples were processed. Following demineralization, the resulting products were washed and neutralized with distilled water and are referred to as tilapia demineralized bone matrix (tDBM) in this study. Subsequently, the samples were transferred to an ultralow-temperature (ULT) refrigerator (Haier, Qiangdao, China) and stored at −80 °C for a minimum of 24 h before undergoing lyophilization. Lyophilization was performed using a freeze dryer (Gyrozen, Gimpo, Republic of Korea) at −55 °C under vacuum for another 24 h. The lyophilized tDBMs were characterized using different histological, physical, and thermal analyses and were compared to a native tilapia bone sample.

### 2.2. Histological Staining

The histological staining protocol was adapted from a previous study [23] with minor modification. Briefly, the tDBM and native tilapia bone samples (around 5 × 5 mm^2^) were initially fixed by soaking in 10% buffered formalin for a period of 3 days and, subsequently, washed with water. The samples were dehydrated with an increasing concentration of ethanol (70%, 95%, 100%) for a period of 1 h per concentration, followed by immersion into xylene for 30 min. The samples were embedded in a paraffin wax to create 2 cm × 2 cm blocks. After embedding, the blocks were sliced into 4 µm thick ribbons using a microtome (SLEE medical GmbH, Nieder-Olm, Germany). The ribbons were then placed on albumin–glycerol fixative-coated glass slides and incubated at 45 °C. Then, the samples were deparaffinized and dehydrated by soaking in xylene and in increasing ethanol concentrations, respectively. Afterward, a hematoxylin and eosin (H&E, Biognost^®^, Zagreb, Croatia) standard protocol [24] was used to stain the samples. Finally, the samples were imaged using a CX22 laboratory microscope (Olympus, Tokyo, Japan) to identify the presence of any visible intact nuclei and the structure of the tDBM.

### 2.3. Infrared Spectroscopy Analysis

Fourier Transform Infrared Spectroscopy (FTIR) provides molecular-level insights that enable the analysis of functional groups, bonding types, and molecular conformations [25] of both native bone and tDBM. Infrared spectroscopy analysis was performed using an IRTracer-100 FTIR (Shimadzu, Kyoto, Japan) to investigate the chemical composition of the samples. The lyophilized samples were positioned onto the spectrum plate and scanned over a wavelength range spanning from 400 to 4000 cm^−1^. The absorbance of infrared wavelengths was automatically quantified by the software and displayed as a percentage in the Attenuated Total Reflectance-Fourier Transform Infrared (ATR-FTIR) spectrum. The background was subtracted from the obtained spectra, and different peaks corresponding to the type I collagen and hydroxyapatite markers were identified.

### 2.4. Thermal Degradation and Denutaration Profile

#### 2.4.1. Differential Scanning Calorimetry (DSC)

The denaturation and melting points of the samples were investigated through differential scanning calorimetry by using DSC 4000 (Perkin Elmer, Waltham, MA, USA). The lyophilized samples were initially size reduced to powdered form using a mortar and pestle. The powdered bone samples, weighing approximately 6.0 ± 0.5 mg, were carefully measured into DSC pans, and then placed on the DSC furnace. The samples were heated in an inert environment from 30 °C to 700 °C with a heating rate of 10 °C/min.

#### 2.4.2. Thermal Gravimetric Analysis (TGA)

The thermal stability of the tDBM and native tilapia bones was assessed by performing thermogravimetric analysis using DTG-60H (Shimadzu, Kyoto, Japan). The bone samples were weighed precisely to about 30.0–30.3 mg. The samples were then subjected to increasing temperatures ranging from 20 °C to 700 °C at a rate of 20 °C/min [26], with an air influx of 15 mL/min. The resulting data were graphed by plotting the temperature values (°C, X-axis) against the weight percentage (%, Y-axis) of the bone samples.

### 2.5. Residual Calcium Determination

The quantification of the calcium residual in the tDBM was determined using X-ray fluorescence (XRF) which utilizes the interaction of X-rays with a sample to determine its elemental composition. The samples had initially undergone size reduction using mortar and pestle. Subsequently, the NexCG II XRF (Rigaku, Tokyo, Japan) analyzer was used to assess the residual calcium of the pelletized bone samples.

### 2.6. Kinetics of Demineralization Process

The rate order of the demineralization process was determined by plotting the residual calcium content of the samples against time. Furthermore, ln[Ca] and 1/[Ca] graphs were produced to ascertain the first and second-order rates, respectively, where [Ca] represents the residual calcium content of the demineralized samples [27]. The plot that is most linear indicates the reaction order of the demineralization process.

### 2.7. Protein Quantification

To extract the protein content, the samples were initially powdered and digested using a solution of 0.5 M acetic acid containing 10 mg of pepsin ((Merk, St. Louis, MO, USA). The resulting mixtures were stirred for 48 h to ensure complete protein hydrolysis. Following this, the solutions were subjected to centrifugation at 1670 rcf for 30 min to separate the supernatant from the solids. The recovered supernatants were analyzed using a Qubit Protein Assay Kit (Thermo Fischer Scientific, MA, USA) and read through a Qubit Fluorometer (Thermo Fischer Scientific, MA, USA) to quantify the protein concentrations.

### 2.8. Statistical Analysis

The mean values of the quantitative data were presented as mean ± standard deviation of the mean and were subjected to a one-way analysis of variance (ANOVA) test. A post hoc Tukey HSD test was then conducted to determine whether the treatment exhibited significant differences. The results indicated that there were significant differences among the reported means at a significance level of *p* < 0.05.

## 3. Results

### 3.1. Histological Staining

**Figure 1:**
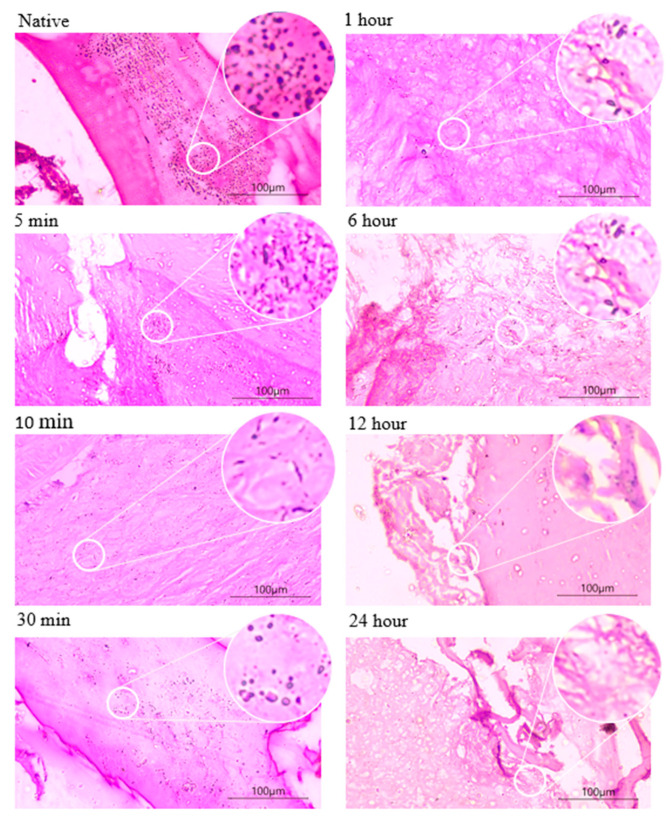
H&E staining images of demineralized bones at different demineralization times with 20× magnification and a scale bar of 100 µm.

The hematoxylin and eosin (H&E) staining of the samples is shown in Figure 1. The native tilapia bone exhibited a significant presence of basophilic components, whereas a gradual disappearance of these components was observed in all demineralized bone samples. The increasing emergence of lacunae or the white gap was observed in 5 min, 10 min, 30 min, and 1 h, and was clearly visible in 6 h, 12 h, and 24 h of demineralization.

### 3.2. Residual Calcium

The results of the residual calcium and protein content are summarized in Table 1, which indicate a progressive decrease in residual calcium over time. The native residual calcium content of the bone was 14.47%, which decreased to 10.09% after 5 min and continued to decrease to 8.72% after 10 min. The decrease in calcium content became more significant with longer demineralization time, as seen by the reduction to 5.43% after 30 min, 1.10% after 1 h, and complete removal after 6 h, 12 h, and 24 h.

Based on the results of the one-way ANOVA, there was a significant difference in the residual calcium among the different durations of demineralization (*p* < 0.001). Post hoc analysis using the Tukey HSD test revealed that all pairwise comparisons of the mean residual calcium were significantly different from each other (*p* < 0.05), except for the comparison between the 6 h and 12 h durations. This suggests that the demineralization duration significantly affects the calcium content, with longer durations resulting in lower residual calcium. The exception for the 6 h and 12 h durations may be due to the very low residual calcium at these durations, which may have reached a minimum threshold.

### 3.3. Kinetics of the Demineralization Process

**Figure 2:**
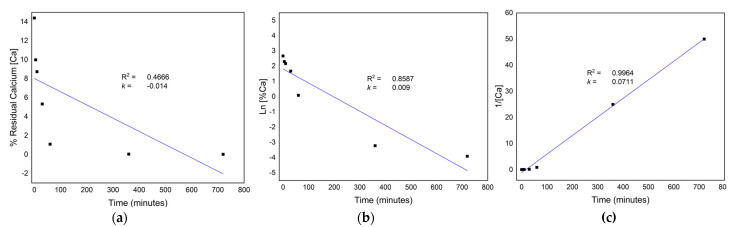
Reaction order of demineralization process with corresponding R^2^ and k value: (**a**) Zero order; (**b**) First order; (**c**) Second order.

The results depicted in Figure 2 suggest that the demineralization reaction follows second-order kinetics with a R^2^ value of 0.9964. Thus, the rate of reaction is proportional to the square of the concentration of the residual calcium as shown in Equation (1).

(1) $$rate=k{\left[Ca\right]}^{2}$$

where *k* is the rate constant of the reaction and [*Ca*] is the residual calcium.

### 3.4. Protein Quantification

Protein is a significant nutrient in fish bones and an important component for nutraceutical applications [28]. The findings indicate a decrease in protein with increasing treatment duration, as illustrated in Table 1 above. Specifically, Table 1 presents a reduction in protein levels from 109.0 ± 1.00 μg/mL to 44.0 ± 1.00 μg/mL after 24 h. The reduction in protein is most evident during the first 30 min of treatment, where it decreased by about 16% from 109.0 ± 1.00 μg/mL at 0 min to 90.7 ± 1.20 μg/mL at 30 min. This suggests that the effect of the treatment on the protein content is very abrupt at the early time intervals than after 6 h.

The one-way ANOVA test results indicate that there is a significant difference in protein content among the demineralization durations (*p* < 0.05). The post hoc Tukey HSD test showed that there were significant differences (*p* < 0.05) in the mean protein concentrations between all time points except for 24 h. These results suggest that the duration of demineralization significantly affects the protein concentration of the bone samples.

### 3.5. Infrared Spectroscopy Analysis

**Figure 3:**
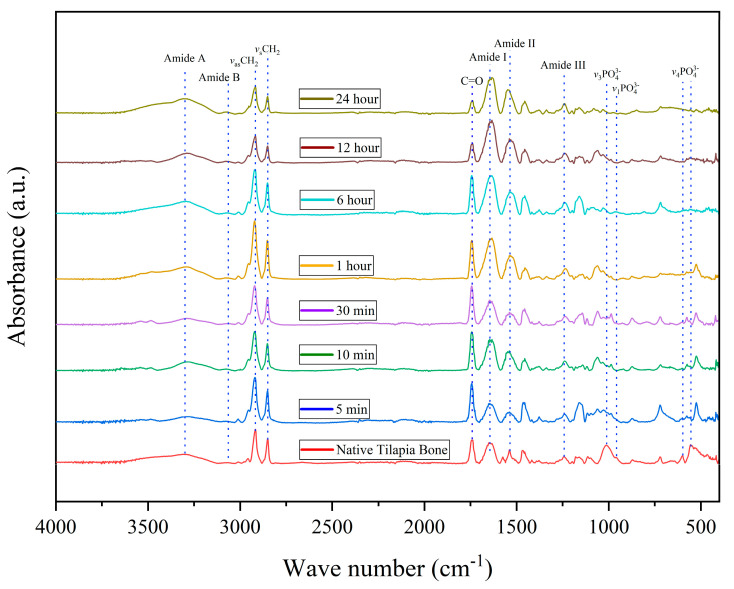
ATR-FTIR spectra of the native and demineralized tilapia bone.

The results depicted in Figure 3 demonstrate that demineralization effectively preserves the collagen type I markers, such as amide I (1640 cm^−1^), II (1537 cm^−1^), and III (1242 cm^−1^), amide A (3300 cm^−1^) and B (3063 cm^−1^), and the symmetric and antisymmetric bands in all demineralized bones. It can also be observed that the intensities of the CH_2_ symmetric (2850 cm^−1^) and antisymmetric (2916 cm^−1^) stretching bands increase with increasing demineralization time, reaching a maximum after 1 h before gradually decreasing. The observed bands in the ATR-FTIR spectra are consistent with previously reported studies, thus confirming that the demineralization process effectively maintains the tilapia bone collagen structure [20]. The peak at 1735 cm^−1^ is associated with the C=O group of peptides in the protein structure and is usually assigned to the carbonyl stretching vibration of amide I [29]. This peak is mainly attributed to the C-terminal regions of a collagen molecule.

The hydroxyapatite peaks can be found in different vibration modes of phosphate, V_4_PO (560 cm^−1^ and 600 cm^−1^), V_1_PO (960 cm^−1^), and V_3_PO (1012 cm^−1^) [30]. The peaks are visible in the native tilapia bone up to 1 h of demineralization but are flattened out in the remaining treatments.

### 3.6. Thermal Degradation and Denaturation Profile

#### 3.6.1. Differential Scanning Calorimetry (DSC)

**Figure 4:**
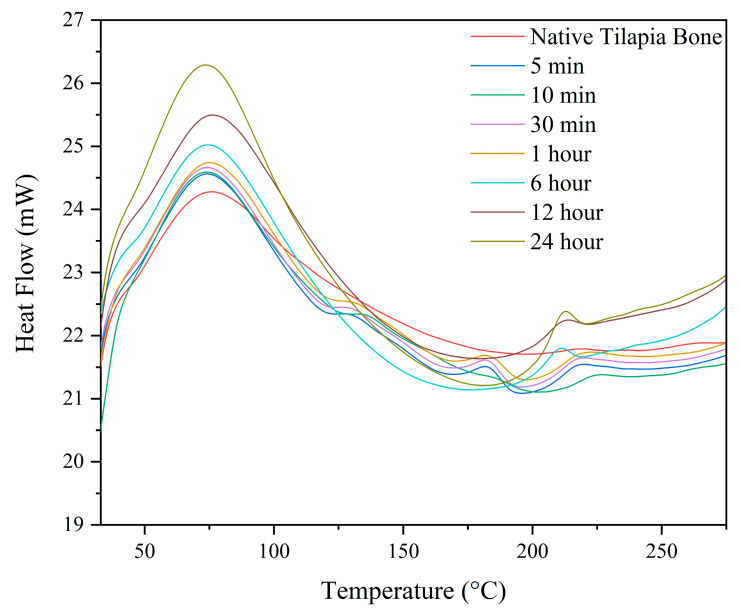
Differential scanning calorimetry (DSC) curve.

The differential scanning calorimetry (DSC) analysis of all the samples conducted in this study revealed two significant endothermal peaks, which are depicted in Figure 4 below. This analysis provides crucial information about the thermal properties of the samples and their underlying molecular structure. The first endothermic peak observed between 70–75 °C represents the thermal denaturation of collagen [31]. The second endothermic peak observed between 210–225 °C represents the complete release of structural moisture, which is responsible for the stability of the triple helix structure of collagen [23]. Additionally, two peaks between 125–130 °C and 180–185 °C were observed that emerged in the demineralized samples at 5 min, 10 min, 30 min, and 1 h.

#### 3.6.2. Thermogravimetric Analysis (TGA)

**Figure 5:**
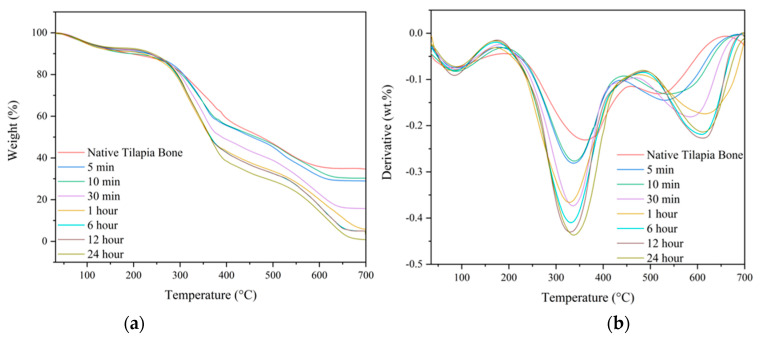
Gravimetric curve of native and demineralized tilapia bone: (**a**) Thermal; (**b**) Differential.

Thermal gravimetric analysis was used to study the thermal degradation of native tilapia bone and tDBM, as shown in Figure 5a. To enhance sensitivity in detecting minor changes in the percent weight loss of the samples relative to temperature, a derivative graph of the thermal gravimetric curve was generated, as illustrated in Figure 5b. It was observed that there were three-step weight loss peaks on the thermal curve for all the treated and native tilapia bone samples as shown also in Table 2. The samples have shown that the first step or the initial degradation peak started from 30 °C to 180 ± 9.0 °C, with corresponding average weight loss of 8.53 ± 0.96 which was attributed to water-loss adsorbed on the surface [30]. The second degradation step occurred at the temperature range from 180 ± 9.0 °C to 466 ± 20.5 °C, with a notable weight loss percentage of 55.67 in 1 h to 62.56 in 24 h, which is attributed to the degradation of collagen and other organic compounds [32]. The third degradation peak occurred at the temperature range from 466 ± 20.5 °C to 687 ± 13.5 °C, which can be attributed to the degradation of residual organic compounds of the bone [33]. The results showed that HCl is efficient in removing the initial mineral content of native tilapia bones at 35%, which decreases as the demineralization duration increases. As depicted in Figure 5a, the mineral content remains relatively consistent at both 6 and 12 h durations, whereas a complete removal of minerals is observed at the 24 h mark, which aligns with the XRF results.

### 3.7. Demineralization Yield

In this study, yield was used to evaluate the effect of different demineralization times. The yield of demineralized bone refers to the percentage of the dry weight of the bone remaining after the demineralization process over the dry weight of native bone as shown in Equation (2).

(2) $$Yield=\;\frac{weight\;after\;demineralization}{initial\;weight}\times 100$$

**Figure 6:**
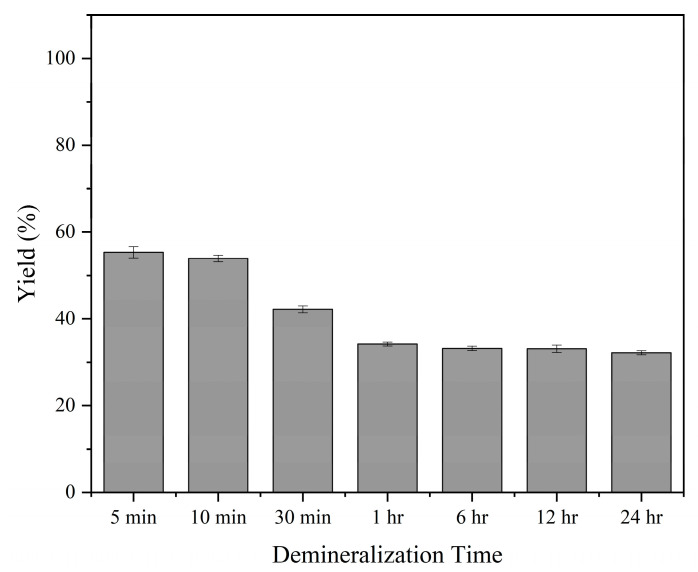
Yield of demineralization with *n =* 3.

From the data presented in Figure 6, the yield of the demineralization process decreased slightly from 56.37 ± 0.46% at 5 min to 53.91 ± 0.75% at 10 min. Further increasing the time to 30 min led to a significant decrease in yield to 42.20 ± 0.80%. After continuing the process for 1 h, 6 h, 12 h, and 24 h, the yields were 34.18 ± 0.44%, 33.17 ± 0.51%, 33.09 ± 0.85%, and 32.18 ± 0.45%, respectively.

## 4. Discussion

Upcycling fish waste, including underutilized resources such as tilapia bones, has recently gained attention due to its potential to create high-value products, promote environmental sustainability, and foster economic growth. By repurposing what would otherwise be discarded, this approach in extracting marine-based extracellular matrix (ECM) not only reduces waste but also creates new opportunities for innovation and market development in the fishing industry.

For many years, demineralized bone matrix (DBM) has been utilized in various forms to treat bone defects. Its osteoconductive and osteoinductive properties make it an invaluable asset in the medical field [34]. At present, very few studies have been carried out regarding the specific effects of prolonged demineralization times on tDBM.

The goal of the study was to investigate the demineralization of tilapia bone using 0.5 N HCl with varying demineralization times ranging from 5 min to 24 h, with the aim of extracting and preserving the extracellular matrix (ECM) structure, mainly collagen type I. Hematoxylin and eosin staining was used to examine the changes in the bone tissue, revealing the gradual disappearance of basophilic components and the emergence of lacunae, which were observed as early as 5 min of demineralization and persisted after 6 h. These changes can be attributed to the removal of mineral content and subsequent decellularization of cells, leaving behind organic components such as collagen. The acid dissolves the minerals in the bone and exposes the organic matrix, making it highly susceptible to acid hydrolysis, leading to the destruction of cell membranes [35].

The results of the study were further validated through residual calcium analysis of the demineralized bones using X-ray fluorescence. The residual calcium content was found to be 1.10 ± 0.12% after 1 h and <0.1% after 6 h of demineralization. Interestingly, allograft bone, which retained 2% of its mineral content after demineralization, exhibited the most significant osteoinductive potential [36,37]. Moreover, understanding the kinetics of the demineralization process is crucial in developing an efficient and effective method for bone demineralization. The second-order rate equation can be used to predict and control the rate of residual calcium removal, which can help to optimize the demineralization process.

The results presented in Table 1 demonstrate that the protein content of the demineralized bone samples decreased gradually as the demineralization time increased. This reduction in protein content is expected, as the demineralization process involves the removal of minerals, including calcium, which is known to bind to collagen [38,39,40]. However, despite the reduction in protein content, key collagen markers were preserved, as shown by ATR-FTIR analysis, indicating that the collagen structure was still maintained in the demineralized bones. Thus, it is likely that the decrease in protein content observed in this study is primarily due to the removal of non-collagenous proteins, while the collagen structure remained intact. Moreover, the spectra did not exhibit any peak shifts, which indicates that the samples did not undergo denaturation[41]. These findings suggest that the demineralization process can effectively extract the mineral content while preserving the structural integrity of the collagen.

The ATR-FTIR spectra analysis provided further evidence that the demineralization process effectively removed hydroxyapatite while preserving collagen markers, including amide I, II, and III, amide A and B, as well as the symmetric and antisymmetric CH2 bands in all demineralized bones. Notably, the intensity of the CH_2_ symmetric and antisymmetric stretching bands increased with longer demineralization time, reaching its peak after 1 h and gradually decreasing afterward. This increase in band intensity can be attributed to the removal of mineral content during demineralization [20,42], which enhances the vibrational movement and flexibility of the ECM. Moreover, as the treatments progressed, a noticeable shift in the V_4_PO peak was observed, which may be attributed to the protonation of hydroxyapatite. These findings suggest that the demineralization process not only effectively removes the mineral content but also enhances the structural properties of the remaining organic components.

The thermal analysis of both native and demineralized bones revealed an increasing weight loss with increasing demineralization time, particularly during the second degradation step. The DSC curve showed additional peaks in the 5 min, 10 min, 30 min, and 1 h durations, likely caused by the evaporation of bound water and the denaturation of calcified collagen [43]. These peaks were present because of residual calcium content in these durations. The thermal analysis provided valuable information on the denaturation temperature and degradation of collagen, which can be related to its stability and functional properties. This is crucial information for the development of applications in biomedical engineering, tissue engineering, and regenerative medicine, where collagen stability and functionality are critical factors for successful implantation and integration with the host tissue.

The demineralization process of tDBM resulted in a decrease in yield as the demineralization time increased due to the removal of minerals and protein degradation. This finding is consistent with Pietrzak et al.’s research, which showed that prolonged acid exposure leads to a decline in residual calcium and bone morphogenic protein levels [22].

The choice of demineralization method is critical in producing high-quality ECM from different sources. Hydrochloric acid (HCl) is a commonly used method for demineralization [44], but the concentration and duration of the acid treatment can significantly impact the chemical composition, microstructure, and mechanical properties of the resulting ECM. To optimize the demineralization process, it is essential to carefully evaluate the impact of different HCl concentrations and treatment durations on the ECM properties. Additionally, the characterization of the resulting demineralized ECM is crucial to ensure its suitability for specific applications.

Using fish bones as a source of extracellular matrix (ECM) can be a cost-effective and readily available alternative to other ECM sources [45]. However, there are several potential limitations and challenges that need to be taken into consideration. Contamination is a significant concern since fish may be exposed to environmental toxins, heavy metals, or other pollutants that can affect the safety and efficacy of the ECM [46,47]. Variability in quality is also a challenge since different fish species and even different parts of the same fish may have different compositions and properties that can impact the ECM’s efficacy. Ethical considerations related to the sourcing of fish bones must also be taken into account, particularly in cases where the fish may be endangered or overfished. To overcome these challenges, it is important to establish quality control procedures and to carefully source fish bones from reputable suppliers. By addressing these challenges, fish-bone-derived ECM can offer a valuable and sustainable source of biomaterials for a range of clinical and research applications.

Scaling up the demineralization process of fish bones to produce ECM at an industrial level and its economic feasibility require careful considerations. Factors such as the cost of raw materials, efficiency of the process, and the potential for environmental impact need to be evaluated. The availability of a steady supply of fish bones, as well as the cost of labor and equipment for processing, must be taken into account. Additionally, the quality and consistency of the final product need to be ensured for commercial viability. Research efforts should focus on optimizing the process to reduce costs and increase efficiency, as well as on exploring potential applications for the extracted bone ECM beyond nutraceuticals, such as in tissue engineering and regenerative medicine.

## 5. Conclusions

The demineralization of tilapia bones has emerged as a promising technique for extracting ECM with potential nutraceutical applications. However, there is currently a lack of research focused on optimizing the demineralization process of tilapia bones while preserving the ECM structure and other important bone components. To address this, the present study investigated the use of 0.5 N HCl for different time durations to demineralize tilapia bones. The study’s findings demonstrate that the demineralization process effectively preserved the ECM structure across all time durations, as confirmed by collagen markers, protein content, and thermal properties. For effective bone ECM extraction, especially collagen extraction, low residual calcium content is desirable to prevent interference with collagen solubility. A residual calcium content of 1% after 1 h of demineralization would be desirable for tissue engineering applications.

This study has provided valuable insights into the demineralization of tilapia bones using HCl. However, it is important to acknowledge that there are some limitations that need to be taken into consideration. Future studies should aim to vary the concentration of HCl and explore other demineralizing agents to establish optimized protocols. In addition, studying the surface morphology and microstructure of the ECM could provide valuable insights into its biological and mechanical properties. Furthermore, to evaluate the protein composition and its possible degradation during demineralization, future confirmatory studies should include techniques such as polyacrylamide gel electrophoresis (PAGE).

## Figures and Tables

**Table 1:** Residual calcium content and protein quantification of the native and demineralized tilapia bone (n = 3).

Duration	Calcium wt %	Protein (µg/mL)
Native	14.47 ± 0.20 *	109.0 ± 1.00 *
5 min	10.09 ± 0.13 *	102.3 ± 1.0 *
10 min	8.72 ± 0.18 *	98.0 ± 0.20 *
30 min	5.43 ± 0.27 *	90.7 ± 1.20 *
1 h	1.10 ± 0.12 *	88.7 ± 0.58 *
6 h	0.04 ± 0.02	51.7 ± 1.52 *
12 h	0.02 ± 0.01	48.7 ± 1.15 *
24 h	n.d.	44.0 ± 1.00

* Significant difference with other groups (*p* < 0.05). n.d. = not detectable.

**Table 2:** Degradation peaks and % weight loss of thermal and differential gravimetric curves.

	1st Degradation		2nd Degradation		3rd Degradation	
Treatment	Temperature, °C	% Weight Loss	Temperature, °C	% Weight Loss	Temperature, °C	% Weight Loss
Native	30–194	10.02	194–459	38.38	459–656	16.6
5 min	30–179	8.66	179–433	39.14	433–686	23.2
10 min	30–181	9.71	181–441	38.31	441–687	36.19
30 min	30–192	8.32	192–468	49.94	468–689	35.54
1 h	30–170	8.68	170–475	55.67	475–692	29.65
6 h	30–175	7.76	175–486	58.61	486–692	28.67
12 h	30–177	7.81	177–486	58.56	486–697	28.67
24 h	30–179	7.24	170–483	62.56	483–700	29.44

## Data Availability

Not applicable.
